# Potential Plant-To-Plant Transmission: Shared Endophytic Bacterial Community Between *Ziziphus lotus* and Its Parasite *Cuscuta epithymum*

**DOI:** 10.1007/s00248-024-02421-z

**Published:** 2024-09-28

**Authors:** Nabil Radouane, Khaoula Errafii, Salma Mouhib, Khadija Ait Mhand, Jean Legeay, Mohamed Hijri

**Affiliations:** 1 African Genome Center, University Mohammed VI Polytechnic (UM6P), Lot 660, Hay Moulay Rachid, 43150 Ben Guerir, Morocco; 2 Institut de Recherche en Biologie Végétale, Département de Sciences Biologiques, Université de Montréal, 4101 Rue Sherbrooke Est, Montréal, QC Canada

**Keywords:** 16S rRNA gene metabarcoding, Arid environment, Complex interaction, *Cuscuta epithymum*, Microbial diversity, Parasitic plant, *Ziziphus lotus*

## Abstract

Microbiota associated with host–parasite relationships offer an opportunity to explore interactions among plants, parasites, and microbes, thereby contributing to the overall complexity of community structures. The dynamics of ecological interactions between parasitic plants and their hosts in arid environments remain largely understudied, especially in Africa. This study aimed to examine the bacterial communities of *Cuscuta epithymum* L. (clover dodder), an epiphytic parasitic plant, and its host, *Ziziphus lotus* L. (jujuba), in an arid environment. Our goal was to uncover the ecological complexities of microbial communities within the framework of plant–plant interactions. We conducted a comprehensive analysis of the bacterial composition and diversity within populations of the *C. epithymum* parasite, the infected- and non-infected jujuba host, and their interface at the shoots of the host. This involved amplicon sequencing, targeting the V5–V6 regions of the 16S rRNA gene. A total of 5680 amplicon sequence variants (ASVs) were identified, with *Pseudomonadota*, *Bacillota*, and *Actinobacteriota* being prevalent phyla. Among the bacterial communities, three genera were dominant: *Cutibacterium*, *Staphylococcus*, and *Acinetobacter*. Interestingly, analyses of alpha-diversity (*p* = 0.3 for Shannon index and *p* = 0.5 for Simplon index) and beta-diversity (PERMANOVA, with *p*-values of 0.6 and 0.3) revealed no significant differences between *Cuscuta*-infected and non-infected jujube shrubs, suggesting a shared shoot endophytic bacteriome. This finding advances our comprehension of microbial communities linked to plant–parasite interactions in the arid environments of Africa. Further research on various hosts is required to confirm plant-to-plant bacterial transmission through *Cuscuta* infection. Additionally, studies on functional diversity, cytology, ecophysiology and the mechanisms by which bacterial communities transferred between host and parasite are necessary.

## Introduction

Parasitic plants are fascinating components of terrestrial ecosystems, influencing ecological dynamics and the structure of plant ecology. Among these parasitic plants, the genus *Cuscuta*, which comprises plant holoparasites belonging to the tribe Cuscutaceae and family Convolvulaceae [1], stands out as an important and economically significant genus known for its detrimental effects on agriculture, especially in sub-humid and semi-arid areas of Africa and Asia [2]. These plants are categorized as obligate parasites because they lack chlorophyll, rendering them unable to perform photosynthesis [3]. Consequently, they rely entirely on their host plants for water, nutrients, and carbohydrates. *Cuscuta* spp. typically exhibit filament-like, yellow to orange stems with flowers that emerge at maturity, entwining around the stems and leaves of their host plants. *Cuscuta* spp. are distributed worldwide, inhabiting various ecosystems from tropical to temperate regions [3]. Due to their parasitic nature, *Cuscuta* spp. are often considered detrimental to agriculture. If not managed effectively, they can significantly reduce the growth and yield of host plants. For example, *Cuscuta* infestations can lead to decreased biomass and reproductive output of crops, ultimately resulting in lower agricultural productivity. However, they also play crucial ecological roles within their ecosystems [4]. Parasitic plants like *Cuscuta* spp. can alter competitive interactions between host and non-host species, affecting community structure and diversity. These plants can influence vegetation zonation and population dynamics by changing the relative abundance of species within plant communities. Furthermore, they can impact other trophic levels, such as herbivores and pollinators, whose behaviors and populations are often closely linked to the presence of parasitic plants. This dual role as both agricultural pests and ecological influencers highlights the complexity of their effects within natural and managed ecosystems [5].

The genus *Cuscuta* encompasses numerous species, including *C. campestris* and *C. epithymum*, known to be the most widespread and aggressive species within this genus [6]. *Cuscuta* spp. initiate their parasitic phase by forming specialized structures called haustoria. These haustoria penetrate the host plant’s stem, providing *Cuscuta* spp. with a physical connection through which they not only extract nutrients but also secure themselves by attaching to the host. As the parasitic relationship progresses, *Cuscuta* spp. continue to proliferate, forming a dense network of intertwining stems that further magnify their impact, eventually leading to detrimental effects on host growth, development, and reproduction [7–9].

*Ziziohus lotus* L. is threatened by habitat loss and degradation due to human activities such as overgrazing and agricultural expansion [10], as well as potential risks caused by *Cuscuta* spp. attacks. Conservation efforts are crucial for protecting this species and preserving its ecological significance in arid environments, especially in the context of global climate change.

In this study, sampling was conducted in the Rhamna region of Morocco, where the *Cuscuta epithymum* species attacks the jujube shrub (*Ziziphus lotus* L*.*), commonly referred to as “*Sedra*”. *Z. lotus* is a resilient species commonly found in arid environments where it forms shrub clusters or patchy vegetation. It is well adapted to survive in harsh conditions with limited water availability and plays a crucial role in ecosystem dynamics. which also forms a deep root system that enables the plant to access water from deep within the soil, allowing it to thrive even in dry conditions. The shrub provides valuable shade and shelter for various arid fauna, contributing to local biodiversity and mitigating soil erosion via wind and water [11]. Furthermore, *Z. lotus* holds a prominent place in traditional medicine due to its various medicinal properties, as well as the ecological services it provides [11–14].

Despite the ecological importance of jujube–*Cuscuta* associations, there is a gap in scientific research regarding their microbial composition and diversity. To date, no studies have specifically investigated the interactions between *Z. lotus* and its parasite *C. epithymum* and their associated microbiota. This represents a significant knowledge gap, considering the pivotal role of microbial communities in mediating plant health, nutrient cycling, and ecosystem functioning. Although a previous study provided insights into the rhizospheric microbial communities associated with both native and cultivated plant species affected by *Cuscuta* parasitism, the influence of host origin and parasitization remains important in shaping these microbial communities and their functions. Specifically, bacterial communities were strongly influenced by the native range of the host plant, whereas fungi were slightly more affected by parasitization [15]; the objective of this study is to document the diversity and community structure of bacteria associated with *Z. lotus* and *C. epithymum*, using amplicon sequencing that targets the 16S rRNA gene [16–18]. This tool has revolutionized the study of microbial diversity, allowing for the rapid and comprehensive characterization of previously unexplored microbial communities in various environments [19].

Through this study, we aimed to advance our understanding of the microbial ecology associated with parasitic plants and their hosts and explore the implications of these interactions with respect to ecosystem dynamics and potential implications for conservation programs. We hypothesize that *Z. lotus* and its parasite *C. epithymum* share an endophytic bacterial community because of the physical connections between their stems via haustoria, which act as a pathway for bacterial transmission between host and parasite. The potential of bacterial transmission between parasitic plants and their host plants occurs solely through physical connections via haustoria may be overly simplistic. While haustoria do facilitate the transfer of nutrients and macromolecules, they are not the only pathway for bacterial transmission [20]. Studies have suggested several alternative mechanisms (reviewed in Pantigoso et al. [21]). For instance, bacterial communities can be influenced by the exchange of volatiles and exudates released into the surrounding environment, which can alter the microbial communities in both the host and the parasite. Additionally, horizontal gene transfer among bacteria in the soil or on the plant surface can result in the sharing of bacterial communities without direct physical connection [20]. To test this hypothesis, we conducted a sampling campaign in arid environments where the jujube shrub naturally thrives and dominates the landscape. Shoot samples were collected from shrubs infected and non-infected by *C. epithymum.*

## Materials and Methods

### Sampling

**Fig. 1:**
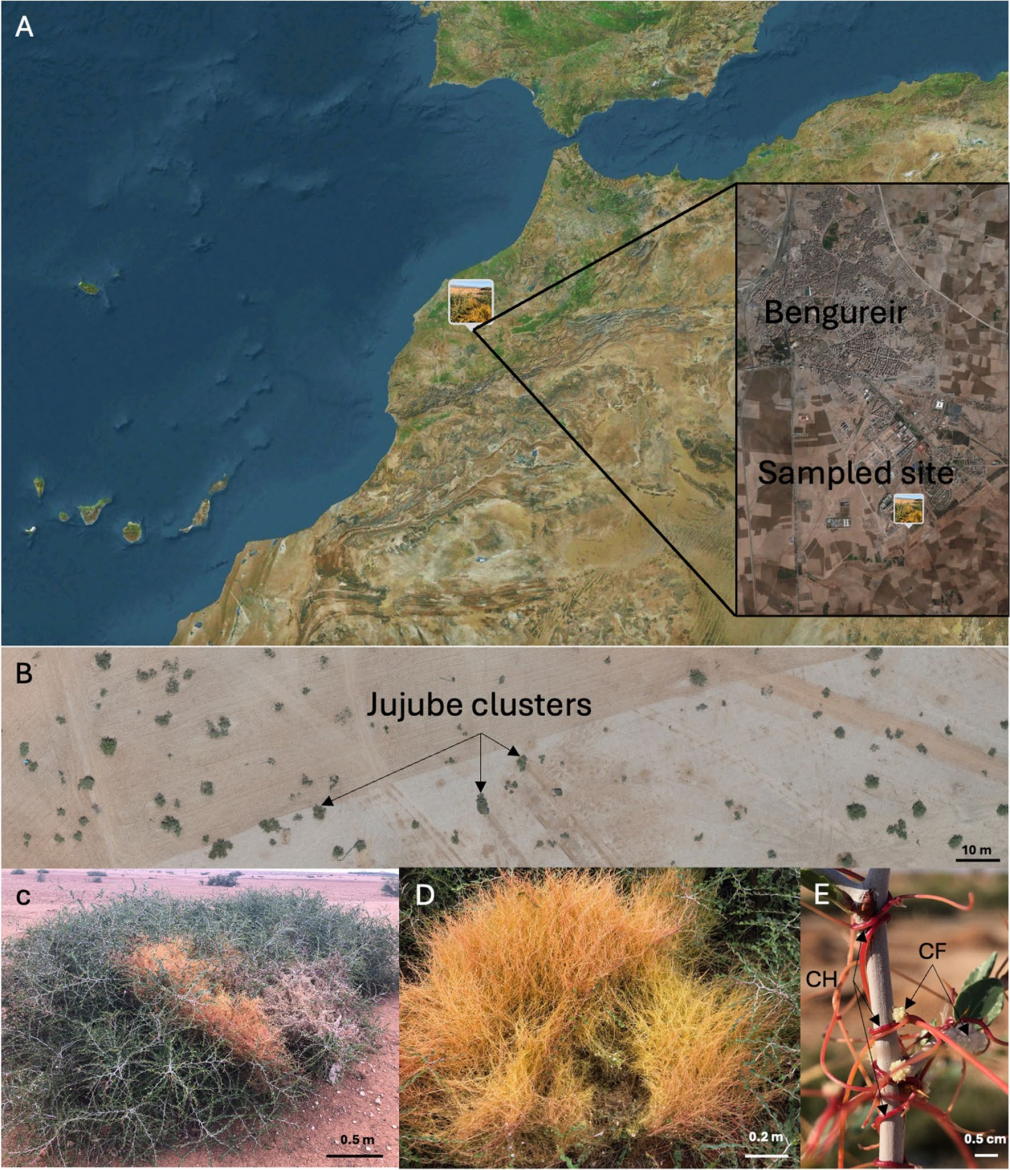
Sample collection from the study area. **A** Map showing the study site located south of Benguerir, Morocco. **B** Aerial view of the site displaying clusters of jujube (*Ziziphus lotus*) shrubs. **C** *Z. lotus* infected with *Cuscuta epithymum* (orange filaments). **D** *C. epithymum* infecting *Z. lotus* shrub. **E** Parasitic relationship between jujube and *C. epithymum*. CF indicates *Cuscuta* flowers; CH indicates *Cuscuta* haustoria

A two-day sampling campaign was conducted in Benguerir, Rhamna, Morocco (N32°12′11.67″, W7°56′8.451″) (Fig. 1A). The sampling took place on June 10 and 11 2020 early morning between 6:00 and 11:00 AM to avoid heat. This sampling period coincided with the flowering stage of *Z. lotus.* The region is characterized by a semi-arid climate with hot, dry summers and mild, wet winters. The soil is predominantly clayey, with low to moderate fertility and low organic matter content, and it is mostly used for growing barley or durum wheat without irrigation. The site features patchy vegetation that is primarily dominated by jujube shrubs, as shown in Fig. 1B. Each patch represents a cluster with a diameter approximately ranging from 1 to 5 m. Nine shrubs of *Ziziphus lotus* L. invaded by *Cuscuta epithymum* were sampled, as illustrated in Fig. 1C. The sample collection involved obtaining young stems (approximately 20 cm long) from various parts of each cluster, including a total of 38 non-infected stems, 39 stems infected by *Cuscuta*, and 38 *Cuscuta* stems alone (yellow to orange stems, Fig. 1D). Additionally, 9 control samples were obtained from a non-infected plant situated away from the infection site (at the periphery of the cluster). Because *Z. lotus* is a thorny plant, thick leather gloves and pruning shears were necessary for collecting samples.

All samples were placed in 20 × 30-cm Ziploc plastic bags, which were then placed on an ice pack and transported to the laboratory (African Genome Center, Benguerir, Morocco). In the laboratory, the non-infected *Z. lotus* stems were processed as follows: From each sample, two to three 5-cm-long top stems were cut; disinfected with 70% ethanol, followed by a bleach solution; and rinsed three times using autoclaved water. They were then placed on autoclaved paper towels for drying. For infected samples (Fig. 1E), only the fragments of *Z. lotus* coiled by *Cuscuta* stems were cut and subjected to the same disinfection, cleaning, and washing procedures as described above. Finally, *Cuscuta* stems were also disinfected, cleaned, and washed. The dried samples were stored in 15-mL containers at − 20 °C until further use.

### DNA Extraction and Quantification

Total DNA extraction was conducted on 50 mg of lyophilized stem fragments from each sample type, including non-infected jujuba, infected jujuba, and *Cuscuta* alone. Samples were ground using a TissueLyser II and 2-mm Tungsten beads (QIAGEN, Global Diagnostic Distribution, Témara, Morocco) in 2-mL tubes for 15 min at a frequency of 24 Hz. The DNeasy Plant Pro kit (QIAGEN, Global Diagnostic Distribution, Témara, Morocco) was used to extract total DNA following the manufacturer’s instructions. The quality and quantity of the extracted DNA were evaluated via gel electrophoresis and DNA quantification with a BioSpectrophotometer (Eppendorf, Hamburg, Germany). During all DNA extraction, PCR and library preparation steps, laboratory safety measures, including the use of vinyl gloves, were followed.

### PCR Amplification

We performed 16S rRNA gene amplicon sequencing, targeting the V5–V6 regions. This approach allows for more specific amplification of bacterial 16S rDNA compared to the V3–V4 regions, thereby minimizing contamination from the plant's chloroplast [22]. The V5–V6 hypervariable region of the 16S rRNA gene from each sample was amplified using the primer pair with custom sequence (CS) adapters at the 5′ end: ***CS1***-799F/***ACACTGACGACATGGTTCTACA-***AACMGGATTAGATACCCKG and ***CS2***-1115R ***TACGGTAGCAGAGACTTGGTCT-***AGGGTTGCGCTCGTTG [23]. PCR reactions were conducted in Biobase PCR cabinet, Model PCR800 (Jinan, Shandong, China). PCR amplification was performed using the Platinum Direct PCR Universal Master Mix (ThermoFisher, Rabat, Morocco) in a final volume of 25 μL. Each PCR contained 1X of the PCR Universal Master Mix, 0.2 µM of each primer, and approximately 10 ng of genomic DNA. The PCRs were run in a thermocycler Mastercycler X50s (Eppendorf, Hamburg, Germany) following this program: initial denaturation at 94 °C for 3 min, followed by 35 cycles consisting of denaturation at 94 °C for 30 s, annealing at 55 °C for 30 s, elongation at 72 °C for 1 min, and a final extension step at 72 °C for 5 min before being held at 4 °C. Each reaction, including negative controls with sterile Milli-Q water and positive controls, was carried out in duplicate.

### Library Preparation and Sequencing

The bacterial 16S rRNA gene amplicon library preparation was generated using Agencourt AMPure XP beads (Beckman Coulter, USA) to clean the PCR products. Two ethanol washes were performed, followed by air drying. Purified PCR products were then resuspended in 10 mM Tris (pH 8.5). A second PCR was performed to attach Illumina sequencing adapters and index tags. PCRs for indexing contained 5 µL of purified PCR product, 2.5 µL of Fluidigm Access Array Barcode 384, and 1X KAPA HiFi HotStart ReadyMix (Roche Sequencing Solutions). The PCR volume was 50 µL per reaction and was run under the following conditions: an initial denaturation at 95 °C for 3 min, followed by 8 cycles of denaturation at 95 °C for 30 s, annealing at 55 °C for 30 s, extension at 72 °C for 30 s, and a final extension at 72 °C for 5 min.

The indexed amplicons were subsequently purified using Agencourt ampure XP beads and quantified using a Qubit assay and the DNA HS kit (Thermo Fisher, Témara Morocco). Library quantification, normalization, and pooling were performed following Illumina’s instructions. The bacterial 16S rRNA gene libraries were sequenced on an Illumina MiSeq sequencing instrument (Illumina, Paris, France) using a MiSeq reagent V3 kit (300 cycles of paired-end sequencing).

### Bioinformatics Analysis

The resulting fastq files were processed using R version 4.3.3 (the R Project for Statistical Computing). The quality profile of the reads was inspected using the DADA2 pipeline implemented in R [24], and the raw sequence reads with poor average quality scores (< 30) were discarded.

The bacterial reads were filtered and trimmed using DADA2 to eliminate primer and adaptor sequences. In addition, error rates for each consensus quality score were evaluated.

The denoised forward and reverse reads longer than 10 bp were merged into a multiple sequence alignment using the DECIPHER package, and amplicon sequence variances (ASVs) were obtained.

Taxonomic annotation was performed using the most updated and extensive SILVA database for bacteria for the resulting amplicon sequence variants (ASVs) [25, 26].

### Statistical Analyses

To normalize the sequencing depth, the ASV abundances in each sample were converted into compositional data using the “*transform*” command of the phyloseq package with the “compositional” parameter [27].

Bacterial alpha diversity was calculated through the Shannon and Simpson indices at the ASV level using the *phyloseq* package [27]. Beta diversity was assessed by computing the Bray–Curtis distance across different microbial taxa, and it was tested through PERMANOVA using the adonis command of the *vegan* package [28]. The command betadispers from the vegan package was also used to test the difference in dispersion between different conditions.

The hub taxa of the communities were determined using the SpiecEasi [29] and igraph package of R [30] by calculating the betweenness of each taxon. The betweenness represents the number of times an ASV is present on an edge connecting other ASVs. Thus, it indicates a probability that the organism corresponding to this ASV mediates interactions in the community.

Random forest models were created using the *randomForest* package of R [31] and 100 repeated trees. The best models created with this package were then evaluated in terms of the strength of their predictive power with respect to discriminating between conditions based on communities; the best predictor taxa were listed. Significant associations of taxa with respect to the conditions were calculated using the *indicspecies* package [32].

## Results

### Taxonomic Composition

**Fig. 2:**
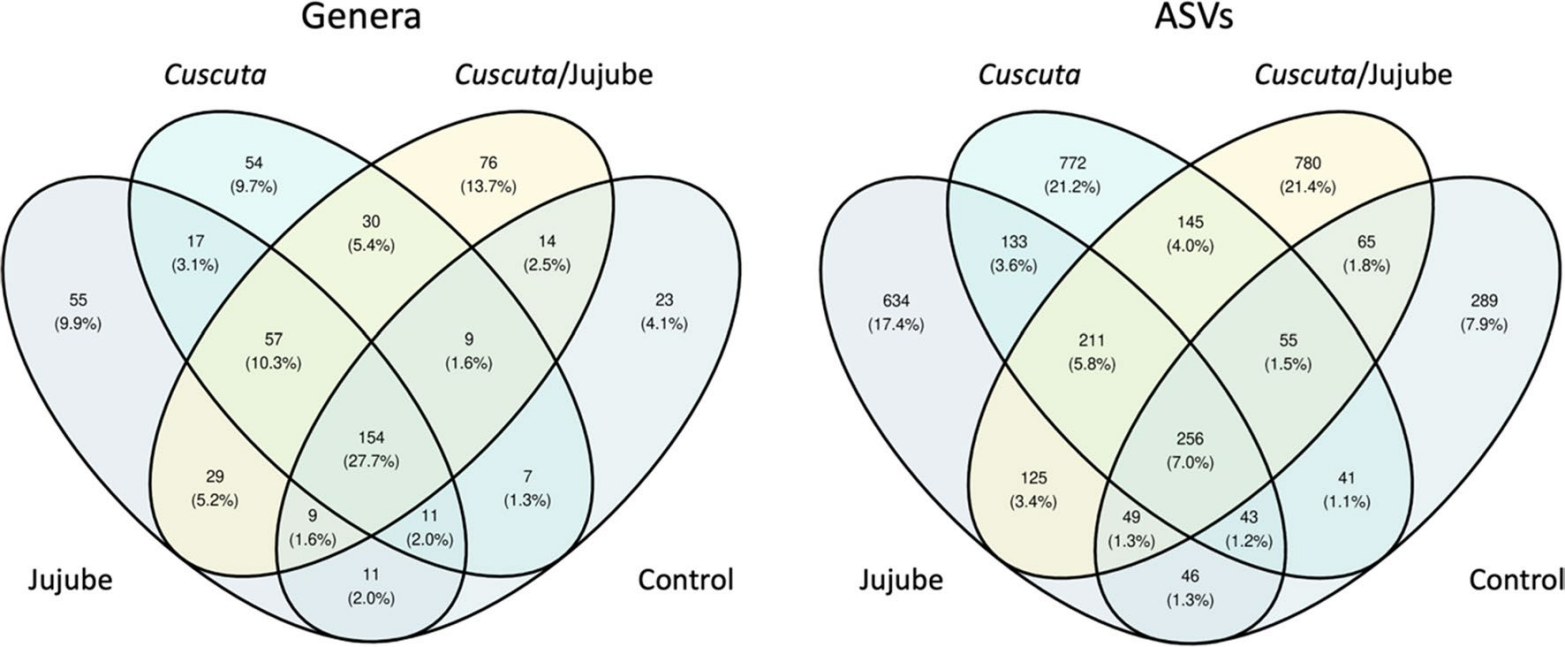
Venn diagrams illustrating the taxa present in various biotopes categorized by **A** genera and **B** ASVs. “Control” refers to non-infected jujube shrubs; “Jujube” refers to *Cuscuta*-infected jujube shrubs; “*Cuscuta*” refers to *C. epithymum* filaments; and “*Cuscuta*/jujube” refers to *Cuscuta* haustoria on jujube stems

**Fig. 3:**
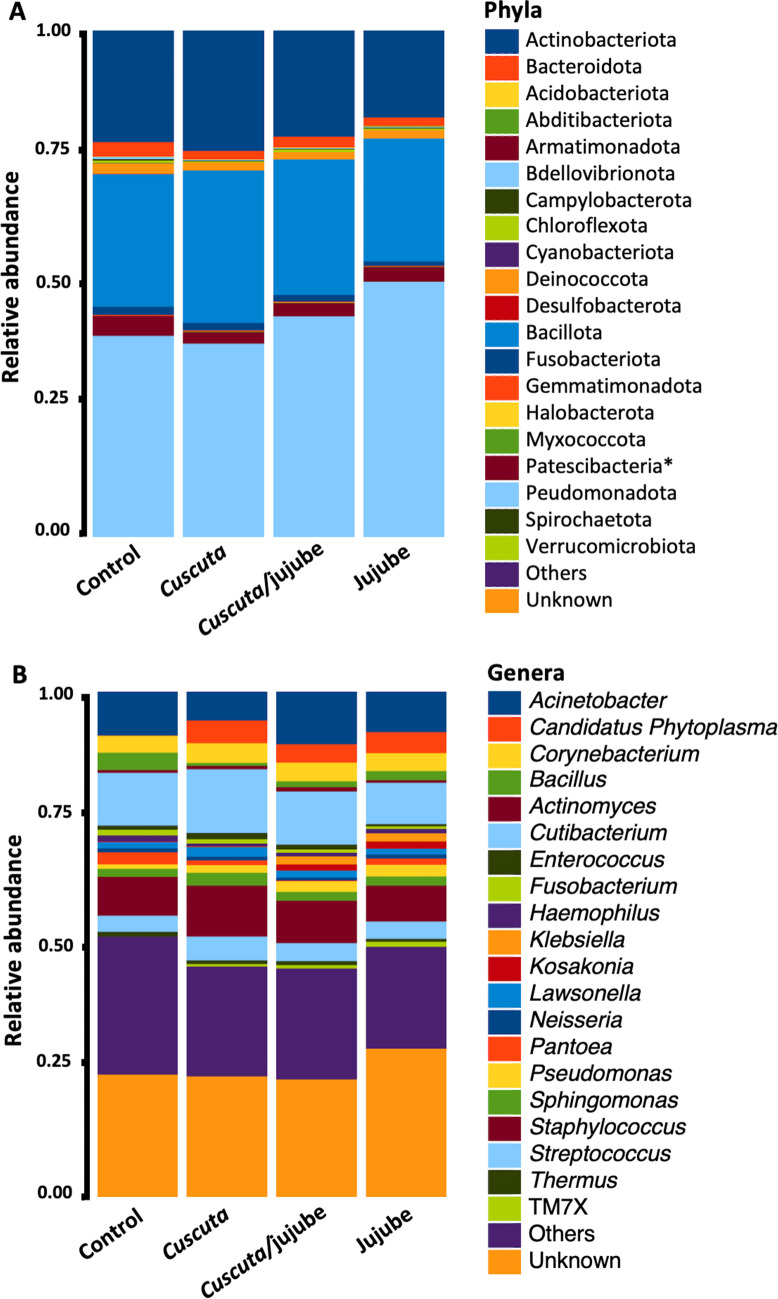
Distribution of the 20 most prevalent taxa categorized by **A** phyla and **B** genera. Taxa not listed among the top 20 most common are grouped under “Others”. * indicates that Patescibacteria is not classified as a phylum but rather as a candidate phyla radiation group. “Control” refers to non-infected jujube shrubs; “Jujube” refers to *Cuscuta*-infected jujube shrubs; “*Cuscuta*” refers to *C. epithymum* filaments; and “*Cuscuta*/jujube” refers to *Cuscuta* haustoria on jujube stems

A total of 1,323,845 reads were detected (approximately 125,000 reads per sample), which were then assigned to 3644 bacterial ASVs comprising 652 genera, 245 families, 134 orders, 62 classes, and 27 phyla. In total, 1947 ASVs were present in jujube samples (Non-infected samples), 1656 ASVs were present in *Cuscuta* samples, and 1686 were present in interaction samples (Infected samples) (Fig. 2). Among these ASVs, 22% were only found in *Cuscuta*, and 29.8% were only found in jujube. The most abundant phyla in all the samples were *Pseudomonadota* (44%), *Bacillota* (27%), and *Actinobacteriota* (21%) (Fig. 3). The most abundant genera were *Cutibacterium* (10%), *Staphylococcus* (8%), and *Acinetobacter* (8%), with a substantial number of ASVs of unknown genus (25%).

### Alpha and *Beta* Diversities

**Fig. 4:**
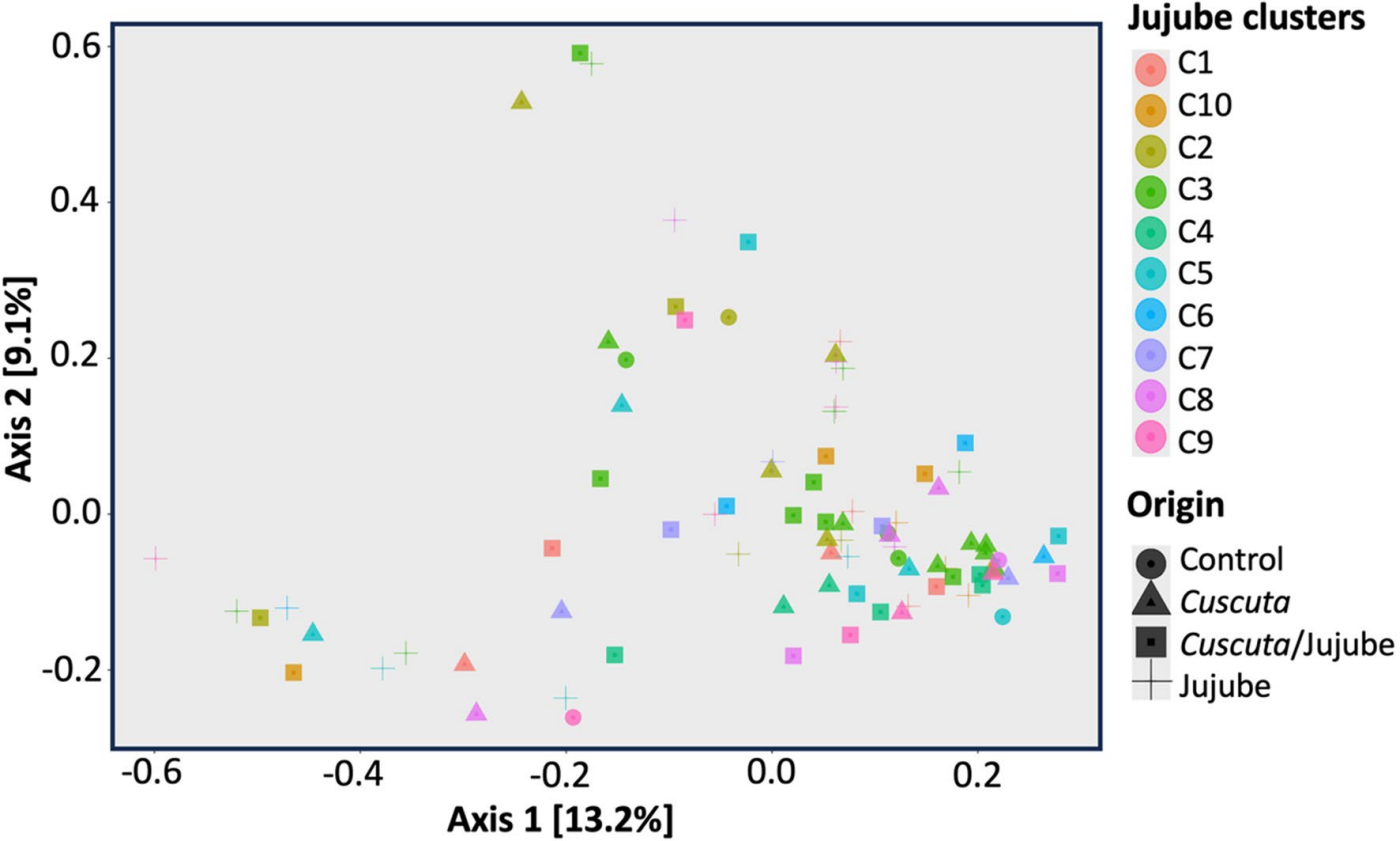
Principal coordinate analysis (PCoA) plot illustrating the samples C1 to C10 of jujube clusters, categorized by their biotopes. “Control” refers to non-infected jujube shrubs; “Jujube” refers to *Cuscuta*-infected jujube shrubs; “*Cuscuta*” refers to *C. epithymum* filaments; and “*Cuscuta*/jujube” refers to *Cuscuta* haustoria on jujube stems

For alpha diversity, both the Shannon and Simpson indices showed no significant differences when comparing origins (Control, *Cuscuta*, *Cuscuta*/Jujube, and Jujube) or samples (with respective *p*-values of 0.3 and 0.5). Additionally, clustering did not significantly influence the Shannon and Simpson indices (with respective *p*-values of 0.1 and 0.4). There were also no significant differences in beta diversity observed either according to the origin or the cluster (PERMANOVA, with respective *p*-values of 0.6 and 0.3). However, a *betadispers* test revealed significant differences in dispersion among communities within certain clusters (*p* = 0.047), although no significant differences in dispersion were found due to the origin (*p* = 0.3) (Fig. 4 and Table 1).

**Table 1:** PERMANOVA test of the phylum using the Aitchison method

	Degrees of freedom	Sum of Sqs	*R*^2^	*F*	Pr(> *F*)
Cluster	9	725.3854	0.1030068	1.020762	0.454
Residual	80	6316.7252	0.8969932	NA	NA
Total	89	7042.1106	1.0000000	NA	NA

### Core Taxa

**Fig. 5:**
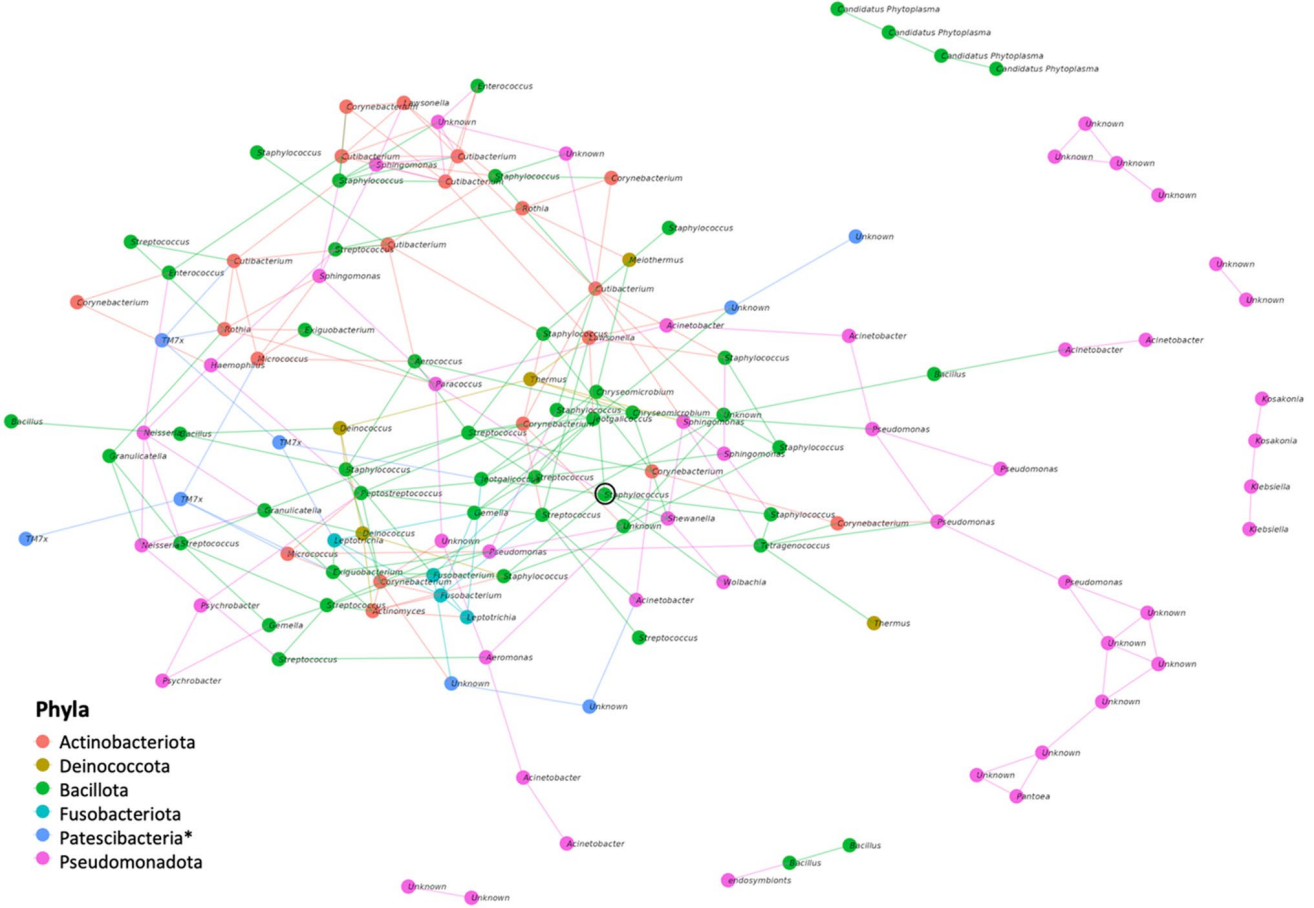
The network betweenness analysis revealed that one ASV from the *Staphylococcus* genus had the highest score among all taxa, indicating its role as a hub ASV

Two ASVs—ASV18 from the *Staphylococcus* genus and ASV21 from the *Cutibacterium* genus—were present in over 90% of all samples. Therefore, these two ASVs can be considered core taxa associated with *Ziziphus lotus* and jujube-infecting *Cuscuta epithymum* in our sampling. Additionally, three other ASVs were found in six out of seven control *Ziziphus lotus* samples: ASV20 from the *Staphylococcus* genus, ASV22 from the Neisseriaceae family, and ASV26 from the *Staphylococcus* genus. At the family and genus levels, the only core taxa detected were those containing the ASV18 and ASV21 mentioned earlier; thus, no additional core taxa were identified at higher taxonomic levels. Following an analysis of network betweenness, one ASV from the *Staphylococcus* genus exhibited the highest betweenness score (1534) among all taxa and can be described as a hub ASV (Fig. 5). Furthermore, as no other ASV has a betweenness score exceeding 95% of this value, it can be considered the sole hub taxon within the community.

### Associated and Predictor Taxa

**Fig. 6:**
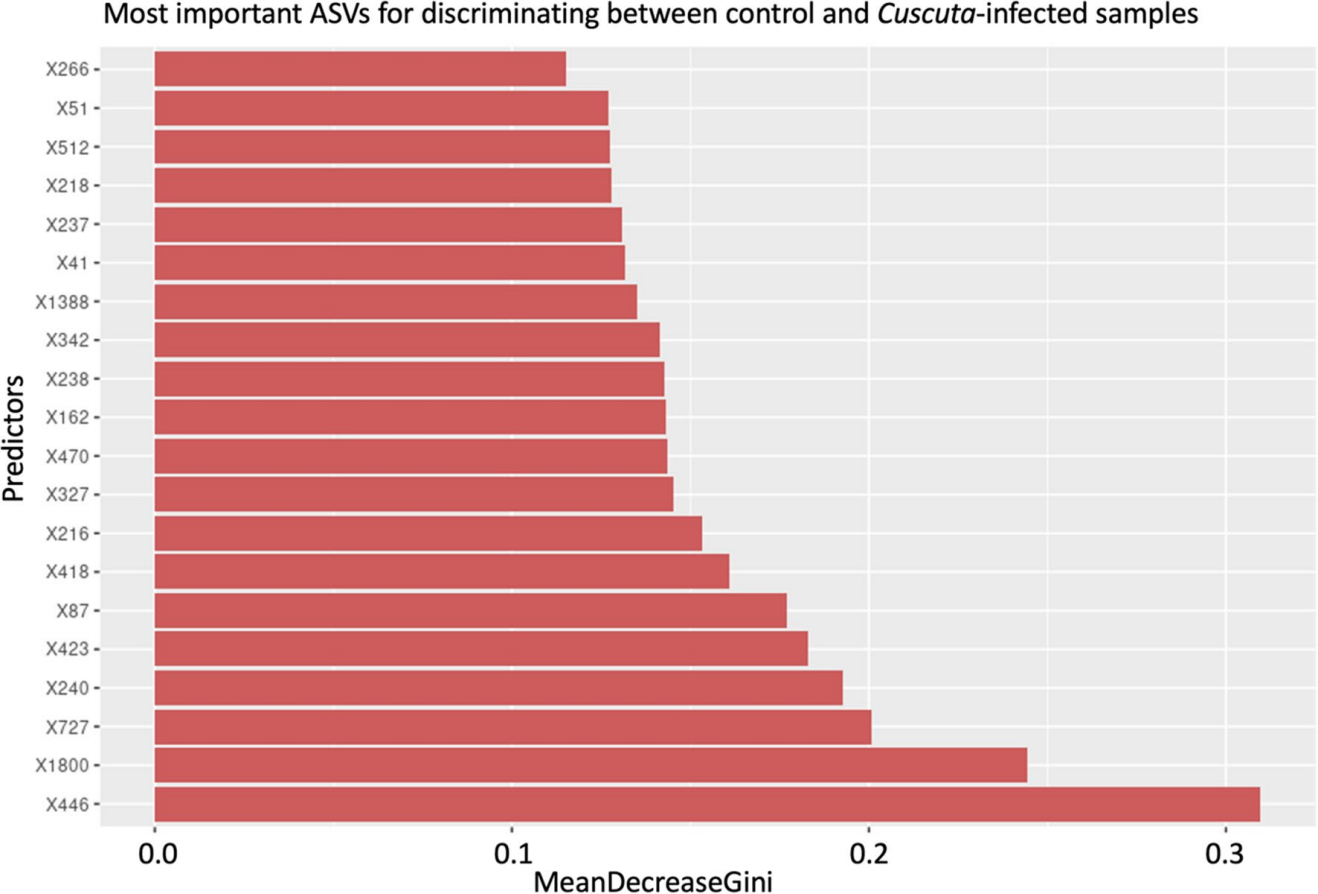
GINI index calculated for the 20 most predictive taxa for distinguishing between control and infected samples, as determined via a random forest model

Interestingly, 40 ASVs from 29 genera (Table 2) were found to be significantly associated with control shrubs (non-infected jujube shrubs away from the infection by *C. epithymum*), whereas infected shrubs had only one ASV from the genus *Caulobacter*, which was significantly associated with their group. No taxa showed differential associations with *C. epithymum*, infected jujube shrubs, or the interaction zone. A random forest model successfully discriminated between control and non-control samples with an error rate of 8.05% (Fig. 6). The 20 most important ASVs for predicting differences between control and non-infected samples are detailed in Table 3. Among these predictor ASVs, four belonged to the *Cutibacterium* genus, three to the *Staphylococcus* genus, and one to the *Acinetobacter* genus.

**Table 2:** Genera significantly associated with the control trees

Genus	Stat	*p*-value
*Acinetobacter*	0.412	0.003**
*Actinotignum*	0.308	0.042*
*Aggregatibacter*	0.386	0.020*
*Aliicoccus*	0.378	0.014*
*Alloprevotella*	0.332	0.022*
*Altererythrobacter*	0.368	0.016*
*Bacillus*	0.415	0.005**
*Blautia*	0.385	0.008**
*Caulobacter*	0.308	0.022*
*Chungangia*	0.297	0.036*
*Corynebacterium*	0.356	0.019*
*Deinococcus*	0.408	0.015*
*Enteractinococcus*	0.316	0.046*
*Escherichia-Shigella*	0.399	0.007**
*Fusobacterium*	0.303	0.031*
*Geodermatophilus*	0.369	0.013*
*Haemophilus*	0.381	0.011*
*Kocuria*	0.408	0.015*
*Methylobacterium-Methylorubrum*	0.281	0.035*
*Microvirga*	0.329	0.017*
*Neisseria*	0.395	0.017*
*Pantoea*	0.272	0.022*
*Peptoniphilus*	0.398	0.017*
*Peredibacter*	0.361	0.029*
*Porphyromonas*	0.373	0.013*
*Streptococcus*	0.271	0.050*
*Streptomyces*	0.323	0.028*
*Tannerella*	0.332	0.033*
*Weissella*	0.364	0.022*

*Significant

**Highly significant

**Table 3:** The 20 taxa with the highest predictive power for distinguishing between control and infected samples

ASV_ID	Phylum	Class	Order	Family	Genus
ASV_9	Pseudomonadota	Gammaproteobacteria	Enterobacterales	Erwiniaceae	Unknown
ASV_14	Pseudomonadota	Gammaproteobacteria	Pseudomonadales	Moraxellaceae	*Acinetobacter*
ASV_18	Bacillota	Bacilli	Staphylococcales	Staphylococcaceae	*Staphylococcus*
ASV_20	Bacillota	Bacilli	Staphylococcales	Staphylococcaceae	*Staphylococcus*
ASV_23	Actinobacteriota	Actinobacteria	Propionibacteriales	Propionibacteriaceae	*Cutibacterium*
ASV_26	Actinobacteriota	Actinobacteria	Propionibacteriales	Propionibacteriaceae	*Cutibacterium*
ASV_29	Pseudomonadota	Gammaproteobacteria	Burkholderiales	Neisseriaceae	Unknown
ASV_30	Actinobacteriota	Actinobacteria	Propionibacteriales	Propionibacteriaceae	*Cutibacterium*
ASV_36	Actinobacteriota	Actinobacteria	Propionibacteriales	Propionibacteriaceae	*Cutibacterium*
ASV_44	Actinobacteriota	Actinobacteria	Corynebacteriales	Corynebacteriaceae	*Lawsonella*
ASV_45	Actinobacteriota	Actinobacteria	Corynebacteriales	Corynebacteriaceae	*Corynebacterium*
ASV_48	Proteobacteria	Alphaproteobacteria	Sphingomonadales	Sphingomonadaceae	*Sphingomonas*
ASV_52	Bacillota	Bacilli	Staphylococcales	Staphylococcaceae	*Staphylococcus*
ASV_56	Bacillota	Bacilli	Lactobacillales	Enterococcaceae	*Enterococcus*
ASV_58	Actinobacteriota	Actinobacteria	Corynebacteriales	Corynebacteriaceae	*Corynebacterium*
ASV_169	Actinobacteriota	Actinobacteria	Micrococcales	Micrococcaceae	*Micrococcus*
ASV_197	Pseudomonadota	Gammaproteobacteria	Enterobacterales	Aeromonadaceae	*Aeromonas*
ASV_200	Actinobacteriota	Actinobacteria	Actinomycetales	Actinomycetaceae	*Actinomyces*
ASV_430	Bacillota	Bacilli	Lactobacillales	Carnobacteriaceae	*Alloiococcus*
ASV_480	Pseudomonadota	Alphaproteobacteria	Rhodobacterales	Rhodobacteraceae	*Paracoccus*

## Discussion

Investigating bacterial diversity within the interaction of *Cuscuta epithymum* and *Ziziphus lotus* through a metabarcoding approach is crucial for obtaining a thorough understanding of the intricate dynamics of plant microbiota. This becomes especially significant given the scarcity of research in this domain, which impedes our comprehension of microbiomes [33].

Among the identified phyla, Pseudomonadota, Bacillota, and Actinobacteriota were the predominant taxa in the bacteriome of both the jujube shrubs and their parasite *C. epithymum*. Together, these phyla constituted the majority of bacterial sequences in the samples. Pseudomonadota was the predominant phylum, comprising 44% of the bacterial community, followed by Bacillota at 27% and Actinobacteriota at 21%. These results align with previous research, which has highlighted the prevalent presence of bacterial taxa such as Pseudomonadota, Acidobacteriota, and Actinomycetota in the phyllosphere of plants thriving in hyper-arid environments[34]. Pseudomonadota, in particular, serves as a notable example of a copiotrophic bacterial phylum, as highlighted in various studies [35–37].

Moreover, two ASVs—ASV18 belonging to the *Staphylococcus* genus and ASV21 belonging to the *Cutibacterium* genus—were found in more than 90% of all samples of this study. ASV18 is a core taxon; it was found to be phylogenetically related to *Staphylococcus hominis*, which has been characterized as a plant endophyte of jute seeds and exhibited antimicrobial activity through antibiotic (homicorcin) production [38]. Staphylococci, frequently associated with humans and recognized for their potential pathogenicity, have been consistently found in plant environments. Notably, metabarcoding studies have revealed their presence as endophytes in seeds, such as those of *Anadenanthera colubrina*, a legume tree [39], and rice [40]. Furthermore, they have been identified in various plant tissues, including soybeans [41].

The genus *Cutibacterium* genus, identified as ASV21, has been reported in *Citrus limon* seeds and shoots [41]. This genus has also been observed as an endophyte in the cultivated grapevine *Vitis vinifera*, comprising 5% of the total sequence reads [42]. Furthermore, the majority of the genera identified in this study from the shoot microbiota are known to exhibit plant-growth promotion activities, such as phosphate solubilization, siderophore production, organic matter mineralization, and nitrogen fixation [43–45]. However, the presence of a high proportion of unknown genera highlights the limitations of the metabarcoding method [46]. These unknowns may represent novel taxa or taxa that are poorly characterized in existing databases, underscoring the need for further investigation and refinement of taxonomic databases.

Furthermore, we identified 40 ASVs from 29 genera (Table 2) that were significantly associated with control shrubs not invaded by *Cuscuta*. This suggests that this community was not homogenized or influenced by the parasitism between jujube and *Cuscuta*.

This study on the endophytic bacteriome of *C. epithymum* and its host plant *Z. lotus* revealed that both partners in the parasitic relationship share a common bacteriome. Microbial species may experience either promotion or inhibition due to the parasitic relationship, thus resulting in diversity affected by plant parasitism [15]. Shifts in the prevalence of certain taxa related to parasitism may impact plant performance. For example, the reduction in the abundance of identified species could affect plant stress tolerance [47, 48]. *Cuscuta* parasitism has also been reported to influence the expression levels of genes essential for bacterial survival and sporulation [49, 50]. In contrast to previous findings suggesting a high degree of host specificity within the microbiota of *Cuscuta pedicellata* and its host plant [51], our results indicate a lack of discernible variation in bacterial communities among the studied samples. The diversity and composition of this shared bacteriome did not exhibit any significant differences or distinct groups among the analyzed samples, including the control. These results indicate that the parasitic relationship between *C. epithymum* and *Z. lotus* does not alter the composition of the *Z. lotus* bacteriome. Instead, they share the same endophytic community, supporting our hypothesis, which posited that *Z. lotus* and its parasite *C. epithymum* share an endophytic bacterial community due to the physical connections between their stems via haustoria, which act as a pathway for bacterial transmission between host and parasite.

While previous studies have identified distinct bacterial and fungal species as endophytes and epiphytes, our findings challenge the notion of host specificity within the phyllosphere microbiota associated with *C. pedicellata* and its host plants [52]. Similarly, the study on rhizospheric microbial communities associated with native and cultivated plant species affected by *Cuscuta* parasitism found that the parasitic relationship did not significantly alter the overall composition of the host plant's bacteriome. However, it emphasized the importance of the host plant’s native range and parasitization in shaping microbial communities and their functions. Bacterial communities were more strongly influenced by the host plant’s native range, while fungal communities were slightly more affected by parasitization [15].

*Cuscuta* spp. establish direct connections with the vascular system of their host plants, including the xylem and phloem [53]. The linkage with the host’s phloem has been demonstrated through experimental findings. For instance, studies using the phloem-specific dye carboxyfluorescein have shown movement from the host into *Cuscuta* tissues [54, 55]. Additionally, research on transgenic tobacco plants expressing green fluorescent proteins in companion cells has indicated the potential transfer of proteins to *Cuscuta*, implying the possibility of direct macromolecule transfer [56]. In our sampling, the infection of *C. epithymum* extended across a vast area, encompassing numerous shoots of various individuals within the cluster (Fig. 1C, D). Hence, it is probable that *C. epithymum* shoots acted as a transmission vector, thereby homogenizing the endophytic bacteriome of *Z. lotus*, including both infected and control samples. However, cytological studies are needed to confirm the extent of haustoria penetration in jujube tissues. A well-documented phenomenon is the transmission of viruses between the host and *Cuscuta* spp. It has been observed that a single *Cuscuta* plant parasitizing two hosts simultaneously may facilitate the transmission of plant viruses from one host to the other [53]. Apart from these direct vascular connections, *Cuscuta* also exhibits cytoplasmic continuity with its host through plasmodesmata [57].

The interaction between *Cuscuta* spp. and its host plants involves complex mechanisms that significantly impact the bacterial communities associated with the host. *Cuscuta* spp., being obligate stem holoparasites, form direct connections to the vascular bundles of their hosts via haustoria, enabling the transfer of water, carbohydrates, and other solutes. This physical connection facilitates the bidirectional exchange of macromolecules such as RNAs [58], which may play a role in signaling between the host and the parasite [58]. The similarity in bacterial communities between *Cuscuta* and its host can be attributed to this intimate vascular connection, allowing not only the exchange of nutrients and solutes but also potentially shared metabolites and signaling molecules. In addition to physical connections, *Cuscuta* and its hosts may exchange compounds that influence their microbial communities [8]. The parasitic interaction often weakens host plants, making them more susceptible to secondary infections by microbes, insects, and nematodes. This increased susceptibility can lead to convergence in the bacterial communities [59], as both the parasite and the host are exposed to similar environmental pressures and microbial communities. Furthermore, *Cuscuta* spp. manipulate the host’s defense mechanisms by releasing susceptibility triggers, including phytohormones and other yet-unknown signals. These triggers are perceived by host receptors, leading to changes in the host's gene expression and susceptibility-related responses. Such physiological changes can create a microenvironment conducive to specific bacterial communities that are shared by both the parasite and the host [8].

*Cuscuta* spp. introduce significant challenges in agricultural settings due to their broad host range and non-specific attack patterns. Moreover, studies have confirmed the capability of *Cuscuta* species to transmit viruses between plants by establishing haustoria connections with the vascular tissues of the host. This method of transmission shares similarities with grafting, yet dodder distinguishes itself by transmitting viruses across distantly related plants, a feat not achievable through grafting, which usually involves closely related species. Furthermore, dodder can passively facilitate virus transmission, particularly under experimental conditions conducive to the movement of nutrients from infected to uninfected plants. Consequently, dodder has been employed in experimental research to transfer viruses from challenging-to-study hosts to more accessible experimental plants [53].

## Conclusion

This study investigated the endophytic bacteriome of *C. epithymum* and its host plant *Z. lotus*, revealing a shared bacteriome between both partners in the parasitic relationship. The diversity and composition of this shared bacteriome did not display any significant differences or distinct groups among the analyzed samples. These findings suggest that the parasitic relationship between *Cuscuta* and *Z. lotus* does not alter the composition of the *Z. lotus* bacteriome; instead, they share the same endophytic community, supporting our hypothesis. *Cuscuta* spp. establish direct connections with the vascular system of their host plants, including the xylem and phloem. Experimental findings have demonstrated the physical connection with the host’s vascular system. In addition to these direct vascular connections, *Cuscuta* also exhibits cytoplasmic continuity with its host through plasmodesmata.

Drawing from this, it is probable that *C. epithymum* plays a role in linking the jujube plants and potentially homogenizing their microbiome, as indicated by the capability to predict control plants using specific ASVs with the random forest model. The isolation and characterization of bacterial endophytes from the shoots of both *Cuscuta* and jujube could aid in designing future investigations to validate the potential transmission of bacterial endophytes between jujube shrubs and its parasite *Cuscuta* using the isolated taxa transformed with fluorescent proteins. Additional studies on various hosts are also needed to confirm plant-to-plant bacterial transmission via *Cuscuta* infection.

## Data Availability

The raw reads corresponding to the control, infected and non-infected jujube as well as Cuscuta samples were submitted to NCBI (https://www.ncbi.nlm.nih.gov/) with accession numbers provided under the BioProject ID: PRJNA1111547.
